# Supplementary material for: Quantifying fluorescent glycan uptake to elucidate strain-level variability in foraging behaviors of rumen bacteria
Source: Microbiome. 2021 Jan 22;9:23. doi: 10.1186/s40168-020-00975-x (PMC7825182; doi:10.1186/s40168-020-00975-x)
Supplement: Supplementary file 2 — Additional file 1. Supplementary figures [file 40168_2020_975_MOESM2_ESM.pdf]

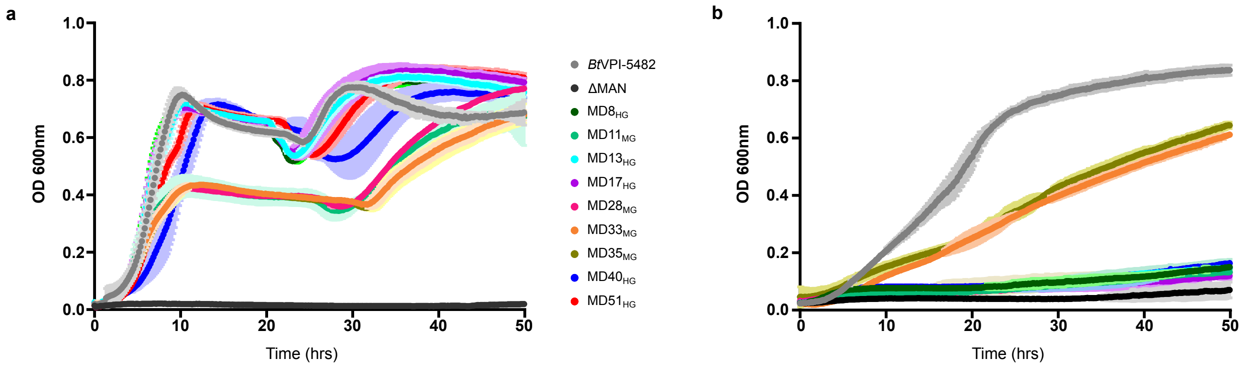

Supplemental Figure 1, Klassen and Reintjes et al., 2020

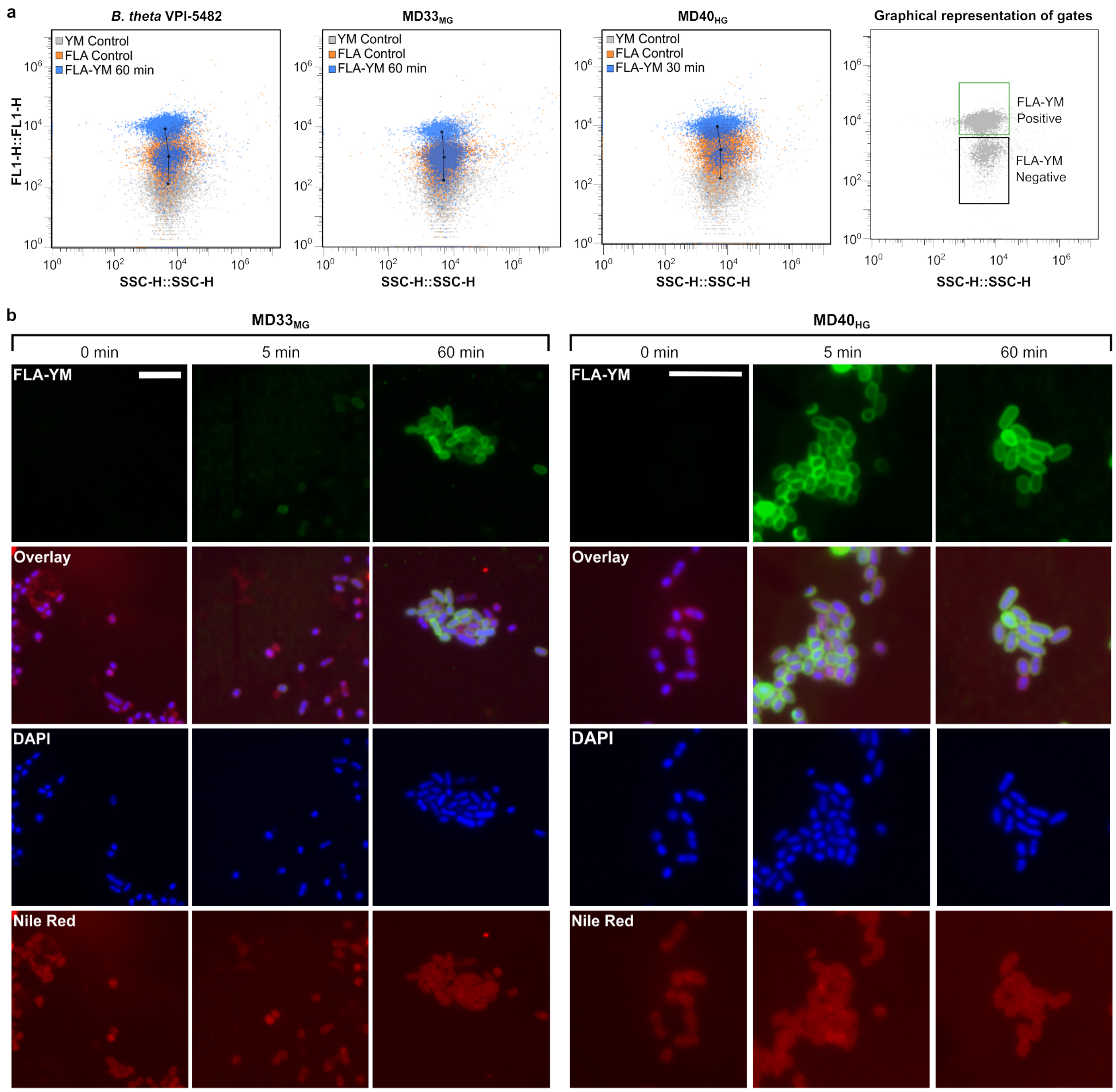

Supplemental Figure 2, Klassen and Reintjes et al., 2020

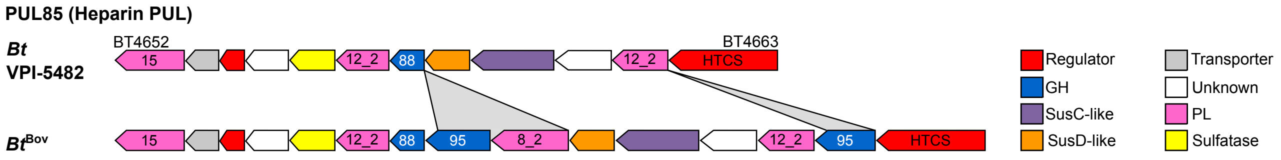

Supplemental Figure 3, Klassen and Reintjes et al., 2020

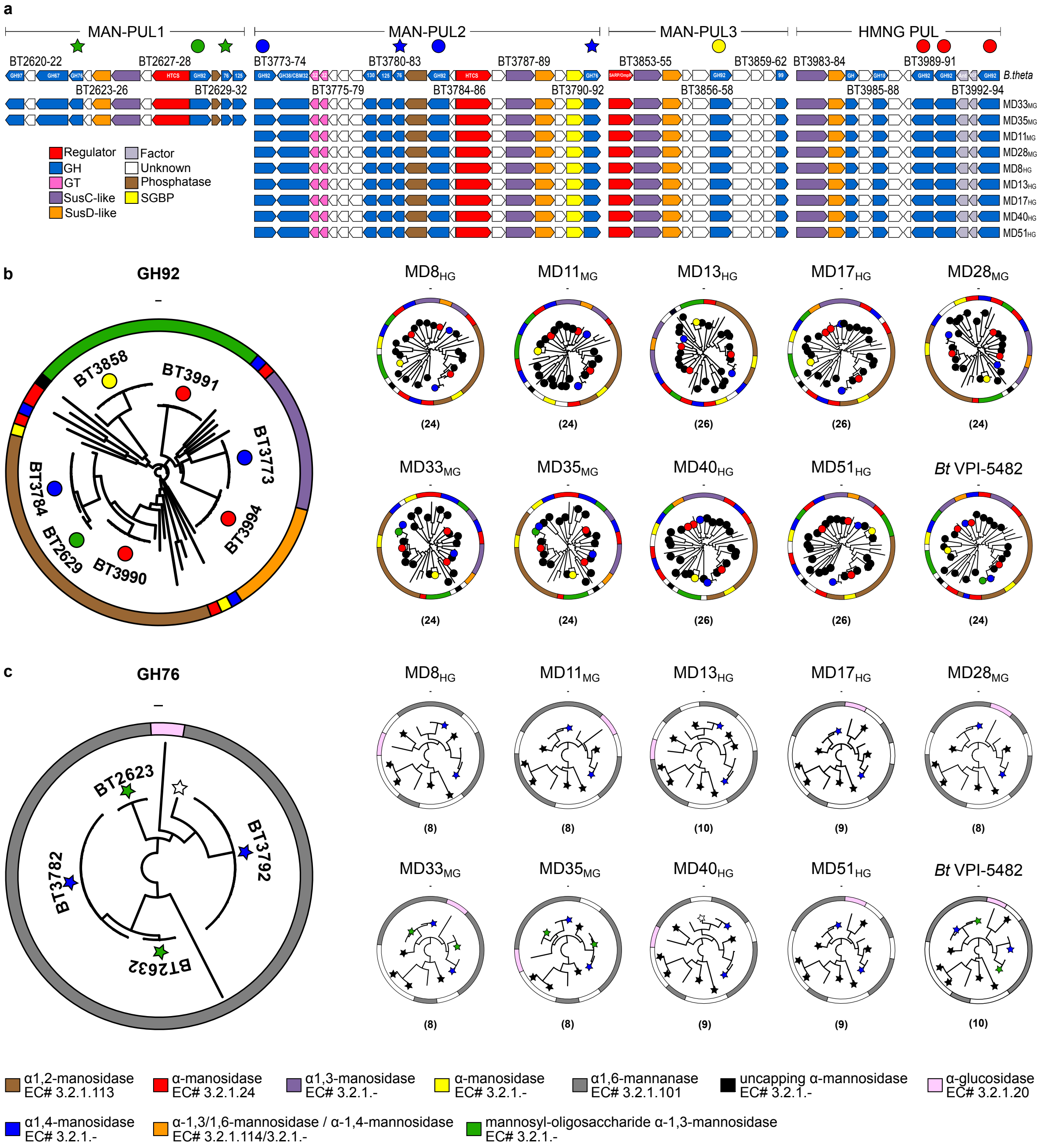

Supplemental Figure 4, Klassen and Reintjes et al., 2020

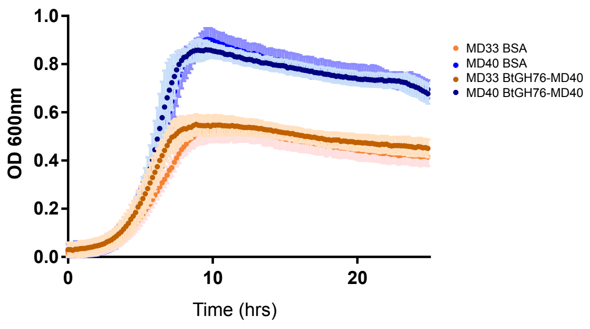

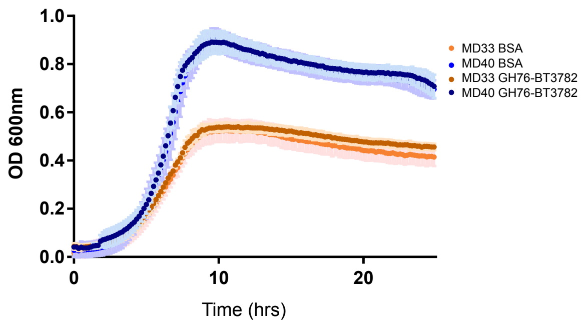

Supplemental Figure 5, Klassen and Reintjes et al., 2020

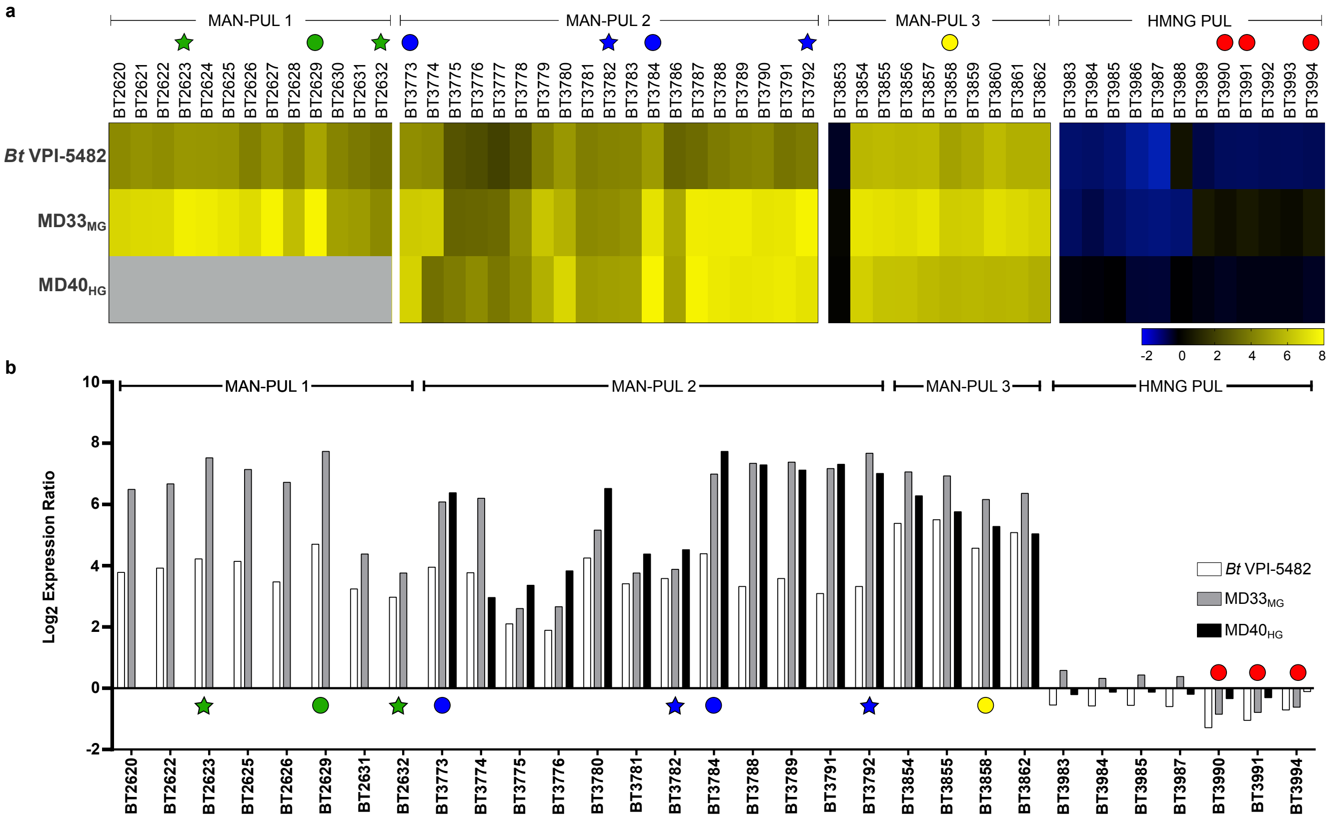

Supplemental Figure 6, Klassen and Reintjes et al., 2020

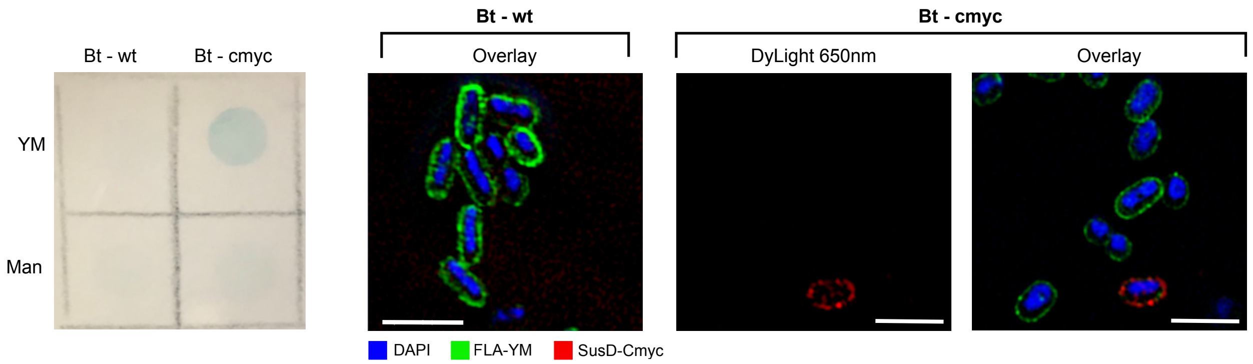

Supplemental Figure 7, Klassen and Reintjes et al., 2020

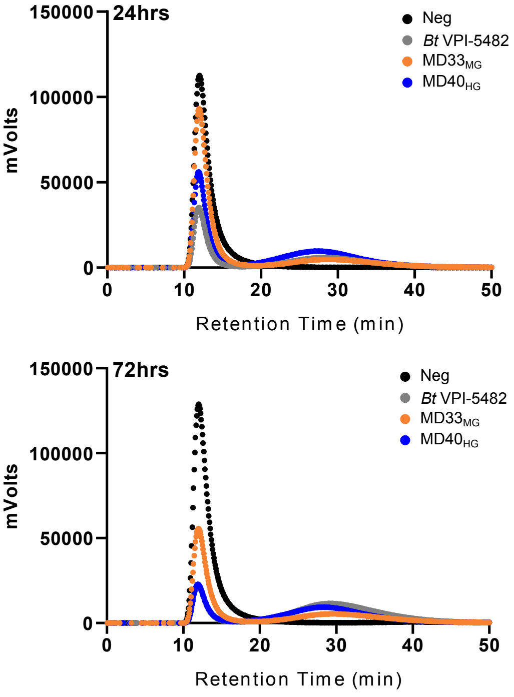

Supplemental Figure 8, Klassen and Reintjes et al., 2020
