# Supplementary material for: Quantifying fluorescent glycan uptake to elucidate strain-level variability in foraging behaviors of rumen bacteria
Source: Microbiome. 2021 Jan 22;9:23. doi: 10.1186/s40168-020-00975-x (PMC7825182; doi:10.1186/s40168-020-00975-x)
Supplement: Supplementary file 3 — Additional file 2. Supplementary materials [file 40168_2020_975_MOESM3_ESM.pdf]

Supplementary Discussion

Catabolite repression in *B. theta* when grown on YM.

In addition to MAN-PUL activation, regions of gene repression were observed in each strain (Fig. 5a). *Bt*VPI-5482 down-regulated (log_2 fold-change < -3.5) many genes when grown on YM, including PUL22, which is predicted to target dietary fructans (*BT_1758-1763*)[1, 2]. This response was observed in $MD33_{MG}$ and $MD40_{HG}$ for a homologous cluster of fructan-utilizing genes (log_2 fold-change < -3.5), suggesting there may be a YM-catabolite repression effect with these two polysaccharides, as it was not observed when the cultures were grown on mannose. In MAN-PUL2, *BT_3775-BT_3776*, which encodes a biosynthetic gene cluster, displayed the lowest expression levels of all the MAN-PUL genes. This is consistent with what was reported previously [3]. Overall, the $MD33_{MG}$ transcriptome had more plasticity in the genes that were either induced or repressed in comparison other two strains (Fig. 5a).

Expression levels of MAN-PULs

Within each MAN-PUL, the *gh*, *susC*-like, and *susD*-like gene clusters consistently displayed the highest log_2 expression values (Fig. 5b, Supplementary Fig. 6). The BT_3788 (*susC*-like, 5.4) and BT_3789 (*susD*-like, 5.5) genes from MAN-PUL3 in *Bt*VPI-5482, BT_2629 (*gh92*, 7.7) and BT_3792 (*gh76*, 7.7) genes in $MD33_{MG}$, and BT_3784 (*gh92*, 7.7) gene in $MD40_{HG}$ displayed the highest expression of all genes studied (Supplementary Fig. 6a).

Host adaptation of *Bt*^{Bov} strains

We observed the presence of signature CAZymes that suggested these strains have adapted to colonize the bovine gut. For example, in every *Bt*^{Bov} strain, two GH95s and two polysaccharide lyases from Family 8 are inserted into the host-glycan utilization locus PUL85, which differs from

the human-associated *Bt*VPI-5482 and 7330 strains (Supplementary Fig. 3). If such host-bacterium interactions are factors required for host colonization, these properties may be exploited for developing bovine-adapted probiotics.

Supplementary Methods

Isolation of bovine-adapted mannan-degraders

Three biological replicates for each sample were incubated anaerobically at 39˚C on a rotary shaker for 24 h. Serial dilutions of the batch cultures (10^0-10^{-6}) were similarly streaked on YM plates. Plates were incubated anaerobically (atmosphere: 85% N_2, 10% CO_2, 5% H_2) at 37˚C for up to 96 h. After 24-96 h of incubation, single colonies were selected and inoculated in nutrient rich Brain Heart Infusion (BHI) medium: 3.7% Bacto™ Brain Heart Infusion (BD; 237500), 8.3 mM L-cysteine, 10mL/L hemin (0.77 mM hemin in 0.01M NaOH), and 0.2% $NaHCO_3$. Overnight cultures were centrifuged and resuspended in 0.8 mL glycerol (50%) and stored at -80˚C.

Growth profiling of bovine isolates

TYG Ingredients: 1% Bacto™ Tryptone (BD; 211705), 0.5% Yeast Extract Bacteriological (VWR; J850), 4.1 mM L-cysteine, 0.2% glucose, 0.1 M KPO_4 pH 7.2, 2.2 µM vitamin K_3, 40 µL/mL TYG Salts (2 mM $MgSO_4 \cdot 7H_2O$, 119 mM $NaHCO_3$, and 34.2 mM NaCl), 28.8 µM $CaCl_2$, 1.4 µM $FeSO_4$, 4.4 µM resazurin, 1 µL/mL (v/v) histidine/hematin (1.9 mM hematin, and 200 mM L-histidine, 1000X stock solution).

Bacteroides MM Ingredients: : 200 mL/L (v/v) 10X Bacteroides salts solution pH 7.2 (999 mM KH_2PO_4, 150 mM NaCl, 85 mM $(NH_4)_2SO_4$), 20 mL/L (v/v) Balch's Vitamins pH 7.0 (36.5 µM ρ-aminobenzoic acid, 4.5 µM folic acid, 8.2 µM biotin, 40.6 µM nicotinic acid, 10.5 µM calcium pantothenate, 13.3 µM riboflavin, 14.8 µM thiamine HCl, 48.6 µM vitamin B_6, 73.8 nM vitamin

B_{12}, and 24.2 mM thioctic acid), 20 mL/L (v/v) Amino Acid Solution (5 mg/mL amino acids: alanine, arginine, asparagine, aspartic acid, cysteine, glutamic acid, glutamine, glycine, histidine, isoleucine, leucine, lysine, methionine, phenylalanine, proline, serine, threonine, tryptophan, tyrosine, and valine), 20 mL/L (v/v) Purine/Pyrimidine Solution pH 7.0 (1 mg/mL adenine, guanine, thymine, cytosine, and uracil), 20 mL/L (v/v) Trace Mineral Solution pH 7.0 (1.7 mM ethylenediaminetetraacetic acid, 12.2 mM $MgSO_4 \cdot 7H_2O$, 3 mM $MnSO_4 \cdot H_2O$, 17.1 mM NaCl, 359.7 µM $FeSO_4 \cdot 7H_2O$, 901.1 µM $CaCl_2$, 347.7 µM $ZnSO_4 \cdot 7H_2O$, 40.1 µM $CuSO_4 \cdot 5H_2O$, 161.7 µM H_3BO_3, 41.3 µM $Na_2MoO_4 \cdot 2H_2O$, and 84.1 µM $NiCl_2 \cdot 6H_2O$), 4.4 µM vitamin K_3, 2.9 µM $FeSO_4 \cdot 7H_2O$, 14.4 µM $CaCl_2$, 2 mM $MgCl_2 \cdot 6H_2O$, 7.4 pM vitamin B_{12}, 16.5 mM L-cysteine, and 2 µL/mL (v/v) histidine/hematin.

Extraction of YM from the cell wall of *S. pombe*

S. pombe was grown in 1 L Yeast Peptone Tryptone (YPD) medium at 30˚C with shaking at 150 rpm for 24 hours. Cells were harvested by centrifugation at 6,000 x g for 10 min. Pellets were resuspended in 10 mL 20 mM Citrate Buffer, pH 7.0 and autoclaved at 125˚C for 90 min. Autoclaved cells were centrifuged at 10,000 x g for 10 min. Supernatant was stored at 4˚C. Pellets were again resuspended in Citrate Buffer, autoclaved, and centrifuged. Supernatants were pooled and mixed with an equal volume of Fehling's Reagent. The solution was incubated at 40˚C, 120 rpm for 2 h. White precipitate formed in the solution and was harvested by centrifugation at 5,000 x g for 10 min. Pellets were dissolved in 2 mL 3 M HCl (per pellet from 1 L of original culture). The solution was slowly transferred to 100 mL of 8:1 Methanol:Acetic Acid with gentle stirring and incubated for 15 min. White precipitate formed in the solution and was collected by centrifugation at 3,200 x g. Supernatant was discarded and the pellet was dissolved in 100 mL 8:1 Methanol:Acetic Acid, vortexed briefly, and centrifuged at 1,500 x g for 5 min. This wash step

was performed two more times and then repeated an additional three times with 100% Methanol. After the final wash, the pellets were left to dry. The following day, the pellets were dissolved in Mq H_2O and dialyzed (500-1,000 MWCO) in Mq H_2O for 20 h. The dialyzed solution was then freeze dried and used for growth analysis.

Genome and 16S rDNA gene sequencing, assembly, and annotation of *Bt*^{Bov} strains

The 16S rDNA was PCR amplified using the following two primers:

Universal primer 27F: 5'-AGR GTT TGA TCM TGG CTC AG-3'

Universal primer 1492R: 5'-GGT TAC CTT GTT ACG ACT T-3'

RNA-seq: assembly, quantitation, and comparative analysis

*Bt*VPI-5482, $MD33_{MG}$, and $MD40_{HG}$ were each inoculated into three tubes containing 5 mL of TYG media. Overnight cultures of *Bt*VPI-5482, $MD33_{MG}$, and $MD40_{HG}$ (OD_{600} 1.0-1.4) were diluted with 2X MM to an OD_{600} 0.05. 100 µL of 1% YM or mannose was added to six wells of diluted culture aliquoted from the same overnight stock, to a final volume of 200 µL. This step was repeated three times for a total of three replicates (each divided among six wells) for each bacterial strain and treatment. Cells were harvested during the exponential phase of the first growth phase (OD_{600} 0.4-0.8). Six wells were pooled and added to an equal volume of RNAprotect (Qiagen). The mixture was vortexed for 5 sec and incubated for 5 min at room temperature. After incubation, the protected cells were centrifuged (10 min; 2,800 x *g*). The supernatant was decanted and pellets were stored at -80°C until further processing.

Production of SusD-like protein C-myc fusion *B. theta* strain

The 5' and 3' regions flanking the *BT_3789* stop codon were amplified by PCR. Primers that contained the linker+C-myc nucleotide sequences were used to amplify the PCR products. These amplicons were then stitched together and ligated into a pEXchange plasmid. The plasmid was transformed into *E. coli* and conjugated into *Bt*VPI-5482 Δtdk Δpul75 recipient strain. Insertion of the C-myc tag was confirmed by sanger sequencing.

Generation of FLA-YM conjugates

To chemically activate the polysaccharide, 350 µL 0.81 M cyanogen bromide (CNBr; 97%; Sigma C91492) was added to 2 mL 2% YM. For ~5 min, pH was monitored and maintained above 9.5 with additions of 0.25 M NaOH. Activated YM was separated from CNBr using Sephadex® G-50 gel filtration medium in a column coupled to a Bio-Rad BioLogic LP Multistatic peristaltic pump (flow rate: 1 mL min^{-1}). The mobile phase consisted of 0.2 M sodium tetraborate decahydrate pH 8.0 (≥99.5%, Sigma S9640). Activated YM was eluted into a vial containing 2.0 mg fluoresceinamine Isomer II (FLA; ~95%; Sigma O7985) wrapped in aluminum foil and incubated for ~24 h at room temperature. To remove excess FLA and purify labelled YM, the reaction mixture was loaded onto Sartorius Vivaspin 15R columns (5,000 MWCO; VS15RH11) and centrifuged (210 x *g*). Columns were repeatedly topped up with distilled H_2O and centrifuged until a clear filtrate was observed. The purified FLA-YM was lyophilized, covered in aluminum foil and stably stored (~4°C) until further use.

Visualization of FLA-YM uptake by strains of *Bt*^{Bov}

For microscopy, samples were resuspended in 1 ml 1 X PBS and, subsequently, 25 µl was heat fixed at 40°C onto a poly-L-lysine coated glass cover slip (12 mm, #1.5H) (Thorlabs GmbH,

Germany). After heat fixation the samples were washed in MQ to remove additional salts and dried at 35°C. The samples were then counter stained with 4', 6-diamidino-2-phenylindole (DAPI) (1 ng µl^{-1} WS) and Nile red (2 ng µl^{-1} WS) for 10 and 25 min, respectively. After each stain, the cells were washed in MQ and subsequently left to dry at RT. Finally, they were mounted onto glass slides using a 4:1 Citifluor (Electron Microscopy Sciences, USA) / VectaShield (Vector Laboratories, Germany) mounting solution.

For epifluorescence microscopy, the samples were visualised using a Zeiss Axioskop 2 motplus fluorescence microscopy with a 100x oil objective. The cells were imaged using the Axiovision software (Zeiss, Germany) and a constant exposure time of 200 ms was applied for the FLA-YM channel (Alexa Fluor 488 filter cube) to enable a comparison of the FLA-YM signal. The subcellular localisation of FLA-YM within individual cells was achieved using a super-resolution structured illumination microscopy (SR-SIM). A Zeiss ELYRA PS.1 microscope with 561, 488, 405 nm lasers and BP 573-613, BP 502-538, and BP 420-480 + LP 750 optical filters was used. Z-stack images were taken with a Plan-Apochromat x63/1.4 oil objective and processed with the ZEN2011 software (Carl Zeiss, Germany).

Supplementary References

1. Martens EC, Chiang HC, Gordon JI: **Mucosal glycan foraging enhances fitness and transmission of a saccharolytic human gut bacterial symbiont.** *Cell Host Microbe* 2008, **4:**447-457.
2. Sonnenburg ED, Zheng H, Joglekar P, Higginbottom SK, Firbank SJ, Bolam DN, Sonnenburg JL: **Specificity of polysaccharide use in intestinal bacteroides species determines diet-induced microbiota alterations.** *Cell* 2010, **141:**1241-1252.
3. Martens EC, Lowe EC, Chiang H, Pudlo NA, Wu M, McNulty NP, Abbott DW, Henrissat B, Gilbert HJ, Bolam DN, Gordon JI: **Recognition and degradation of plant cell wall polysaccharides by two human gut symbionts.** *PLoS Biol* 2011, **9:**e1001221.
4. Altschul SF, Gish W, Miller W, Myers EW, Lipman DJ: **Basic local alignment search tool.** *J Mol Biol* 1990, **215:**403-410.
5. Jones DR, Thomas D, Alger N, Ghavidel A, Inglis GD, Abbott DW: **SACCHARIS: an automated pipeline to streamline discovery of carbohydrate active enzyme activities within polyspecific families and de novo sequence datasets.** *Biotechnol Biofuels* 2018, **11:**27.

S1 Table: Alignment of 16S rDNA gene sequences from mannan-degrading *B. theta* strains isolated from the rumen blasted against the NCBI database[4].

Strain	Top Blast Hit(s)	Query cover (%)	Identity (%)
**MD11**$_{\mathrm{MG}}$	*B. theta* VPI-5482 and strain 7330	100	99.7
**MD28**$_{\mathrm{MG}}$	*B. theta* strain 7330	100	99.7
**MD33**$_{\mathrm{MG}}$	*B. theta* VPI-5482	100	99.8
**MD35**$_{\mathrm{MG}}$	*B. theta* VPI-5482 and strain 7330	100	99.7
**MD8**$_{\mathrm{HG}}$	*B. theta* VPI-5482 and strain 7330	100	99.7
**MD13**$_{\mathrm{HG}}$	*B. theta* VPI-5482 and strain 7330	100	99.7
**MD17**$_{\mathrm{HG}}$	*B. theta* strain 7330	100	99.7
**MD40**$_{\mathrm{HG}}$	*B. theta* strain DMF	100	100
**MD51**$_{\mathrm{HG}}$	*B. theta* strain 7330	100	100

S2 Table: NCBI accession number and assembly parameters of bovine-associated bacterial isolates. SPAdes *de novo* genome assembly output of nine Bt^{Bov} isolates sequenced by Illumina MiSeq PE150bp and ANIb output of Bt^{Bov} assembled contigs blasted to multiple *B. theta* strains from the JSpeciesWS genome reference database.

ACCESSION NUMBER	ISOLATE	SOURCE	SPADES CONTIGS	SPADES LARGEST (BP)	SPADES N50	ANIB STRAIN	ANIB (%)
**SAMN11961934**	MD8$_{HG}$	Rumen	920	868,373	157,167	VPI-5482	98.03
**SAMN11961935**	MD11$_{MG}$	Rumen	62	589,645	236,443	VPI-5482	98.05
**SAMN11961936**	MD13$_{HG}$	Rumen	61	781,280	229,189	KLE1254	99.16
**SAMN11961937**	MD17$_{HG}$	Rumen	59	928,731	208,464	KLE1254	99.15
**SAMN11961938**	MD28$_{MG}$	Feces	63	838,230	236,443	7330	98.32
**SAMN11961939**	MD33$_{MG}$	Feces	72	759,138	150,716	7330	98.33
**SAMN11961940**	MD35$_{MG}$	Feces	70	723,774	145,695	7330	98.33
**SAMN11961941**	MD40$_{HG}$	Feces	62	519,222	189,229	7330	98.34
**SAMN11961942**	MD51$_{HG}$	Feces	62	868,373	229,189	7330	98.33

Number of contigs does not include contigs ≤ 1000 bp.

S3 Table: Statistical significance of RNAseq TPM expression differences between *Bt*VPI-5482, $MD33_{MG}$, and $MD40_{HG}$ MAN-PUL genes when grown in YM.

Strain		TPM	SEM	*B. theta*	MD33	MD40
[1]***B. theta***	BT_2620	279	39		!	-
	BT_2622	720	96		!	-
	BT_2623	252	30		!	-
	BT_2625	660	106		!	-
	BT_2628	635	52		!	-
	BT_2629	2454	385		!	-
	BT_2631	398	64		!	-
	BT_2632	1087	105		ns	-
	BT_3773	179	18		!	!
	BT_3774	604	44		!	!
	BT_3775	1646	30		!	ns
	BT_3776	1832	62		ns	ns
	BT_3780	859	16		!	!
	BT_3781	1103	113		ns	ns
	BT_3782	414	18		ns	ns
	BT_3783	440	52		!	ns
	BT_3784	1834	154		ns	ns
	BT_3786	2594	64		!	ns
	BT_3789	2600	358		!	!
	BT_3791	265	40		!	!
	BT_3792	602	45		!	!
	BT_3853	20	2		!	ns
	BT_3855	12	3		!	ns
	BT_3858	24	4		!	ns
	BT_3862	21	2		!	ns
**$MD33_{MG}$**	BT_2620	13	3	!		-
	BT_2622	16	2	!		-
	BT_2623	24	4	!		-
	BT_2625	804	93	!		-
	BT_2628	183	40	!		-
	BT_2629	2289	219	!		-
	BT_2631	877	46	!		-
	BT_2632	1045	300	ns		-
	BT_3773	1201	134	!		ns
	BT_3774	981	53	!		!
	BT_3775	941	157	!		ns
	BT_3776	1370	38	ns		ns
	BT_3780	1635	142	!		!
	BT_3781	1012	163	ns		ns
	BT_3782	1566	144	ns		!

	BT_3783	1533	75	!		!
	BT_3784	1301	257	ns		ns
	BT_3786	1905	146	!		ns
	BT_3789	132	18	!		!
	BT_3791	352	43	!		!
	BT_3792	279	49	!		!
	BT_3853	449	8	!		!
	BT_3855	1394	45	!		!
	BT_3858	826	102	!		!
	BT_3862	326	23	!		!
	BT_3773	1261	99	!	ns	
	BT_3774	892	83	!	!	
	BT_3775	655	22	ns	ns	
	BT_3776	3754	533	ns	ns	
	BT_3780	1677	104	!	!	
	BT_3781	30	6	ns	ns	
	BT_3782	66	17	ns	!	
	BT_3783	36	6	ns	!	
**MD40$_{HG}$**	BT_3784	239	20	ns	ns	
	BT_3786	870	128	ns	ns	
	BT_3789	237	75	!	!	
	BT_3791	91	12	!	!	
	BT_3792	357	66	!	!	
	BT_3853	107	24	ns	!	
	BT_3855	200	20	ns	!	
	BT_3858	645	128	ns	!	
	BT_3862	163	30	ns	!	

[1]*Bt*VPI-5482.

S4 Table. Percent identity matrix of the MAN-PUL2 SusC/D/E-like amino acid sequences from isolated strains generated by MUSCLE [76].

Strain	MD11$_{MG}$	MD28$_{MG}$	MD33$_{MG}$	MD35$_{MG}$	MD8$_{HG}$	MD13$_{HG}$	MD17$_{HG}$	MD40$_{HG}$	MD51$_{HG}$	*Bt*[1]
**SusC**										
**MD11$_{MG}$**	100	100	100	100	77	77	77	77	77	77
**MD28$_{MG}$**	100	100	100	100	77	77	77	77	77	77
**MD33$_{MG}$**	100	100	100	100	77	77	77	77	77	77
**MD35$_{MG}$**	100	100	100	100	77	77	77	77	77	77
**MD8$_{HG}$**	77	77	77	77	100	100	100	100	100	100
**MD13$_{HG}$**	77	77	77	77	100	100	100	100	100	100
**MD17$_{HG}$**	77	77	77	77	100	100	100	100	100	100
**MD40$_{HG}$**	77	77	77	77	100	100	100	100	100	100
**MD51$_{HG}$**	77	77	77	77	100	100	100	100	100	100
***B. theta*[1]**	77	77	77	77	100	100	100	100	100	100
**SusD**										
**MD11$_{MG}$**	100	100	100	100	81	81	81	81	81	81
**MD28$_{MG}$**	100	100	100	100	81	81	81	81	81	81
**MD33$_{MG}$**	100	100	100	100	80	80	80	80	80	80
**MD35$_{MG}$**	100	100	100	100	80	80	80	80	80	80
**MD8$_{HG}$**	81	81	80	80	100	100	100	100	100	100
**MD13$_{HG}$**	81	81	80	80	100	100	100	100	100	100
**MD17$_{HG}$**	81	81	80	80	100	100	100	100	100	100
**MD40$_{HG}$**	81	81	80	80	100	100	100	100	100	100
**MD51$_{HG}$**	81	81	80	80	100	100	100	100	100	100
***Bt*[1]**	81	81	80	80	100	100	100	100	100	100
**SusE**										
**MD11$_{MG}$**	100	100	99	99	78	78	78	78	78	78
**MD28$_{MG}$**	100	100	99	99	78	78	78	78	78	78
**MD33$_{MG}$**	99	99	100	100	78	78	78	78	78	78
**MD35$_{MG}$**	99	99	100	100	78	78	78	78	78	78
**MD8$_{HG}$**	78	78	78	78	100	100	100	100	100	100
**MD13$_{HG}$**	78	78	78	78	100	100	100	100	100	100
**MD17$_{HG}$**	78	78	78	78	100	100	100	100	100	100
**MD40$_{HG}$**	78	78	78	78	100	100	100	100	100	100
**MD51$_{HG}$**	78	78	78	78	100	100	100	100	100	100
***Bt*[1]**	78	78	78	78	100	100	100	100	100	100

[1] *Bt* = *Bt*VPI-5482. Grey shading represents conservation ≥100%.

Supplementary Figure Legends

Supplementary Fig. 1 Differential glycan utilization of *Bt* strains. Growth profiles of *Bt*VPI-5482, *Bt*ΔMAN-PUL1/2/3, and nine rumen isolates grown on 0.5% YM extracted from the cell wall of **(a)** *S. cerevisiae* (N=4) and **(b)** *S. pombe* (N=3). Mean ± standard deviation shown.

Supplementary Fig. 2 Comparative uptake of FLA-YM by *Bt* strains over time. **(a)** Single cell measurements from pure cultures of *Bt*VPI-5482, $MD33_{MG}$, and $MD40_{HG}$. Grey represents cells incubated with YM-MM. Orange shows cells incubated with unconjugated FLA. Blue indicates cells incubated with FLA-YM for the time noted. Graphical representation of how flow cytometry data was gated, with cells above a certain threshold assigned the FLA-YM positive status. N=10.000. **(b)** Epifluorescence images of $MD33_{MG}$ (left), and $MD40_{HG}$ (right) incubated with FLA-YM for 0 min, 5 min, and 60 min. Cells were counter stained with DAPI and Nile Red. Scale bar is 10 µm.

Supplementary Fig. 3 CAZyme updates to PUL85 in the genomes of the $\boldsymbol{Bt^{Bov}}$ isolates. Grey triangles represent gene inserts into the Bt^{Bov} PUL85-like region relative to *Bt*VPI-5482 PUL85.

Supplementary Fig. 4 CAZyme analysis of GH92 and GH76. (a) Synteny of MAN-PUL1/2/3 and HMNG-PUL of *B. theta* and nine Bt^{Bov} strains. Length of genes are to scale and arrows indicate directionality. GH76s are indicated with coloured triangles, whereas GH92s are indicated with coloured circles. MAN-PUL1 = Green, MAN-PUL2 = Blue, MAN-PUL3 = Yellow, and the HMNG-PUL = Red. **(b)** GH92s and **(c)** GH76s characterized and predicted sequences from Bt^{Bov} genomes generated with SACCHARIS[5]. Large trees (left) show all characterized GH92 or GH76 enzymes from CAZy and those within the *Bt*VPI-5482 genome, as well as the homologous MAN-PUL enzymes from the Bt^{Bov} isolates. Smaller trees display all the characterized GH92 or GH76 enzymes from CAZy, with all of the predicted GH92/76 enzymes found in the genomes of the Bt^{Bov} strain noted above the tree. Outer ring represents characterized specificities (see legend). Circles (GH92) and stars (GH76) represent sequences from PULs: Green = MAN-PUL1, Blue = MAN-PUL2, Yellow = MAN-PUL3, Red = HMNG PUL, and White = PUL55. Numbers in parenthesis indicate the total number of enzymes within each strain.

Supplementary Fig. 5 Growth profiles of *Bt*^{Bov} cultures supplemented with GH76 recombinant enzymes. MD33$_{MG}$ and MD40$_{HG}$ cells cultured in YM-MM supplemented with BSA control protein, *Bt*^{Bov} GH76 from PUL55 (BtGH76-MD40), or GH76 from MAN-PUL2 (GH76-BT3782).

Supplementary Fig. 6 Expression of MAN-PUL gene products in *Bt*VPI-5482, MD33$_{MG}$, and MD40$_{HG}$. (a) Expression levels of MAN-PUL1/2/3 and HMNG-PUL genes. Grey represents gene transcripts not present in the corresponding genome. **(b)** Transcript expression of MAN-PUL1/2/3 in *Bt*VPI-5482, MD33$_{MG}$, and MD40$_{HG}$. Bar graph represents log_2 fold-change of CAZymes and SusC/D/E-like proteins in MAN-PUL1/2/3 and HMNG-PUL encoded in the genomes of *Bt*VPI-5482 (white), MD33$_{MG}$ (grey), and MD40$_{HG}$ (black). GH76 enzymes indicated by stars. GH92 enzymes indicated by circles. MAN-PUL1 = green. MAN-PUL2 = blue. MAN-PUL3 = yellow. HMNG-PUL = red.

Supplementary Fig. 7 Co-localization of epitope tagged SusD membrane protein and FLA-YM staining in *Bt*VPI-5482 cells. Dot blot showing YM-MM (YM) or mannose (Man) cultures of *Bt*Δtdk Δpul75 and a mutant strain with a C-Myc fused SusD-like protein. SR-SIM of *Bt*Δtdk Δpul75 (Bt – wt) stained with DAPI and FLA-YM. SR-SIM of the C-Myc mutant (Bt – cmyc) showing DyLight antibody signal and co-stained with DAPI and FLA-YM. Scales shown are 2 μm.

Supplementary Fig. 8 Product profiles of YM utilization in the supernatants of *Bt*^{Bov} isolates. Chromatograms of hydrolysis products in the supernatants of *Bt*VPI-5482, MD33$_{MG}$, and MD40$_{HG}$ when incubated in FLA-YM for 24 hrs (top) and 72 hrs (bottom).
